# Comparative Analysis of Neurotoxicity of Six Phthalates in Zebrafish Embryos

**DOI:** 10.3390/toxics9010005

**Published:** 2021-01-07

**Authors:** Cong Minh Tran, Trinh Ngoc Do, Ki-Tae Kim

**Affiliations:** 1 Department of Environmental Energy Engineering, Seoul National University of Science and Technology, Seoul 01811, Korea; congtran@seoultech.ac.kr; 2 Department of Environmental Engineering, Seoul National University of Science and Technology, Seoul 01811, Korea; trinh.ngocdo@gmail.com

**Keywords:** neurotoxicity, phthalates, zebrafish, mechanism, neurochemical

## Abstract

The effects and underlying mechanisms of phthalates on neurotoxicity remain unclear as compared with the potentials of these substances as endocrine disruptors. The locomotor activities of zebrafish embryos were investigated upon exposure to six phthalates: dimethyl phthalate (DMP), diethyl phthalate (DEP), benzyl butyl phthalate (BBzP), di-2-ethylhexyl phthalate (DEHP), di-n-octyl phthalate (DnOP), and diisononyl phthalate (DiNP). Moreover, changes in fluorescence intensity in the green fluorescent protein (GFP) transgenic (Tg) lines *Tg(HuC:eGFP)*, *Tg(sox10:eGFP)*, and *Tg(mbp:GFP)* were measured after exposure to six phthalates, and changes in the expression profiles of genes involved in the cholinergic (*ache*) and dopaminergic systems (*dat*, *th*, and *drd1b*) were assessed. Exposure to BBzP, DEHP, and DiNP affected larval behaviors, whereas exposure to DMP, DEP, and DnOP revealed no alterations. A reduced expression of *Tg(HuC:eGFP)* was observed upon exposure to BBzP, DEHP, and DiNP. The expression of *Tg(sox10:eGFP)* and *Tg(mbp:GFP)* was reduced only in response to BBzP and DiNP, respectively. Further, exposure to DiNP upregulated *ache* and *drd1b*. The upregulation of *ache* and downregulation of *drd1b* was observed in DEHP-exposed groups. Exposure to BBzP suppressed *th* expression. These observations indicate that exposure to phthalates impaired embryogenesis of the neurological system and neurochemicals in zebrafish embryos, although the detailed mechanisms varied among the individual phthalates. Further mechanistic studies are needed to better understand the causality between phthalate exposure and neurotoxicity.

## 1. Introduction

Phthalates, or phthalate esters (PAEs) as the esters of phthalic anhydride, are widely used as plasticizers in polymer solutions for manufacturing film coatings [1], cosmetics (e.g., soaps, shampoos, and nail polishes), and various household items (e.g., toys, detergents, and bags) [2,3,4]. PAEs are ubiquitous in the environment and cause exposure to humans via food consumption [5,6], thereby raising concerns of possible risks to human health. Epidemiological studies have reported associations between urinary PAE levels and various health problems, such as childhood obesity, insulin resistance, and asthma [7,8,9].

Several studies have reported that PAEs are endocrine disruptors that dysregulate hormone synthesis, decrease steroid hormone levels, and inhibit steroid hormone receptors [10,11,12,13,14]. Endocrine disruptors have been linked to adverse impacts on the nervous system, as hormone synthesis and feedback are regulated by the hypothalamus and pituitary [15,16]. Several PAEs, including dibutyl phthalate, di-2-ethylhexyl phthalate (DEHP), and diisononyl phthalate (DiNP), are reported to exert negative effects on social and sexual behaviors, and to cause DNA damage in the neural system [17,18,19]. However, it is difficult to compare the relative extent of neurotoxicity induced by PAEs because of variations in test species and targeted PAEs.

Zebrafish embryos are small, transparent, and develop rapidly. In particular, the central nervous system (CNS) and encephalon of zebrafish develop within 3 days post-fertilization (dpf) [20]. Various strategies have been established to quantitatively assess the embryonic and larval behaviors [21,22], as well as to comprehensively evaluate neurotoxicity resulting from exposure to neurotoxic chemicals. In addition, transgenic (Tg) zebrafish have been developed to explore the underlying mechanisms of neurotoxicity [23].

The present study aimed to comparatively assess the neurotoxicity of six PAEs using an embryonic zebrafish model. Based on the occurrences in human and environmental samples [24,25,26], the following six PAEs with low to high molecular weights were selected for assessment: dimethyl phthalate (DMP), diethyl phthalate (DEP), benzyl butyl phthalate (BBzP), DEHP, di-n-octyl phthalate (DnOP), and DiNP. Developmental toxicity testing was initially conducted to determine sublethal concentrations for neurotoxicity analyses. In addition, the effects of these substances on the photomotor behaviors of zebrafish embryos were analyzed with respect to speed (i.e., bursting, cruising, and freezing) and movement parameters (i.e., distance, duration, and count). In addition, alterations in the fluorescence intensity (FI) of three Tg lines after exposure to the six PAEs were measured to explore the mechanisms underlying neurotoxicity. Finally, changes to the expression profiles of transcription factors involved in neurotransmission were measured.

## 2. Materials and Methods 

### 2.1. Zebrafish Husbandry

Wild type zebrafish (*Danio rerio*, AB lines) were raised in a water flow-through system (Zebtec, Tecniplast, Italy) at a constant temperature of 28.5 °C and under a 14/10-h light/dark photocycle. Zebrafish adults were fed with Gemma micro 300 pellets (Skretting, Fukuoka, Japan) three times per day. Two females and one male were isolated in a matting-cage overnight. After the wall was removed the next morning, the fish were allowed to spawn for 30 min. Embryos were collected and healthy embryos were chosen and staged under a microscope as described by Kimmel et al. (1995) [20]. Embryos at the blastula stage, 2–4 h post-fertilization (hpf), were used for PAE exposure. These embryos were incubated in E2 media without methylene prior to exposure. According to the European Union (EU) Directive 2010/63/EU, early-life stages of zebrafish (<120 hpf) are not protected [27,28].

### 2.2. Chemical Exposure and Developmental Toxicity

The following six tested PAEs were purchased from Sigma-Aldrich (St Louis, MO, USA): DMP (CAS: 131-11-3, >99% purity), DEP (CAS: 84-66-2, 99.5% purity), BBzP (CAS: 85-68-7, 98% purity), DEHP (CAS: 117-81-7, powder), DnOP (CAS: 117-84-0, 98% purity), and DiNP (CAS: 28553-12-0, >99% purity). Stock solutions (10 g/L) of each PAE were prepared using 100% dimethyl sulfoxide (DMSO; CAS 67-68-5; >99.5% purity, Sigma-Aldrich). Twelve zebrafish embryos with four replicates were exposed to various concentrations of each PAE (i.e., 0, 0.5, 5, 50, 500, 1000 [1 K], 10,000 [10 K], and 100,000 [100 K] μg/L). PAEs were exposed from 2–4 hpf to 120 hpf. All test solutions were diluted using E2 media and the final concentration of DMSO was adjusted to 0.01% (*v*/*v*). A single embryo was placed in each well of a sterile 96-well plate (Falcon^®^; Corning Incorporated, Corning, NY, USA) containing 200 μL of test solution. From 1 to 5 dpf, 70% of the solution was replaced daily. After exposure to each phthalate, the mortality and malformation were recorded according to a previous study [29]. Screened malformations are defects in body axis, eye, snout, jaw, ear, brain, somite, pectoral, and caudal fin, circulation, trunk, and notochord, abnormalities in pigmentation, swim bladder, and touch response, and edemas in yolk sac and heart. Body length and eye diameter were measured using ToupView (Hangzhou, Zhejiang, China) under a microscope.

### 2.3. Locomotor Response Analysis

To measure larval behavior, zebrafish embryos were exposed to six concentrations of each PAE (i.e., 0, 0.5, 5, 50, 500, 1 K, and 10 K μg/L) prepared with 0.01% DMSO as used in developmental toxicity testing. After exposure from 2–4 hpf to 120 hpf, the locomotor activity of 48 larvae at each PAE concentration was measured using a ZebraBox tracking system (View Point Life Sciences, Inc., Lyon, France) under light and dark transitions: 5 min dark (acclimation), 5 min light, 15 min dark, and 5 min light. The light conditions were applied as described in our previous study [22]. Total swimming distance was calculated by the summation of larval movement collected every 60 s. Based on swimming speed, the number and duration of movements such as bursting (>20 mm/s), cruising (5–20 mm/s), and freezing (<5 mm/s) were calculated for each light/dark/light cycle. All malformed and dead larvae were not used for behavior assessment. Locomotor activity was monitored at the same time each morning.

### 2.4. Tg Zebrafish Larvae Assay

Zebrafish from three green fluorescent protein (GFP) Tg lines, *Tg(HuC*:*eGFP)*, *Tg(sox10*:*eGFP)*, and *Tg(mbp*:*GFP)*, provided by the Zebrafish Center for Disease Modeling (Chungnam National University, Daejeon, South Korea), were acclimated and cultured in the same system for the culture maintenance of the AB line. Three Tg lines were chosen to investigate changes in expression of each target transcription factor (*elav13*, *sox10*, and *mbp*, respectively) that plays a role in the primary motor neurons, neural crest cells, and myelination involved in CNS development. The obtained embryos were exposed to BBzP, DEHP, and DiNP at concentrations of 0, 5, 50, 500, 1 K, and 10 K µg/L under the same conditions (200 μL test solution, 96 well-plate, and exposure from 2 hpf). Then, 10 embryos or larvae from each treatment group were randomly selected for FI measurement using a stereomicroscope system (MZ 10F; Leica Microsystems GmbH, Wetzlar, Germany). GFP intensity was measured at an excitation wavelength of 450 nm and an emission wavelength of 490 nm and quantified using Image J software (v. 1.52p; https://imagej.nih.gov/ij/). FI was averaged from 10 images/concentration/Tg line. To measure changes in FI following exposure to PAE, the relative of FI was normalized to that observed in untreated larvae. Timepoints and regions of FI measurement for the three Tg lines [*Tg(HuC*:*eGFP)*, *Tg(sox10*:*eGFP)*, and *Tg(mbp*:*GFP)*] were determined by the expression of each target transcription factor [30,31,32]. For *Tg(HuC*:*eGFP)*, *elavl3* is highly expressed in the trunk at 48–72 hpf, although expression is masked by thick muscle and most Rohon–Beard neurons undergo apoptosis after 96 hpf [30,31]. Thus, FI was measured in the trunk at 72 hpf. For *Tg(sox10*:*eGFP*), FI was measured in three hemisegments at the end of yolk extension in the trunk at 72 hpf. For *Tg(mbp*:*GFP)*, FI was measured in the region from the forehead to yolk extension at 120 hpf. *Mbp* is associated with myelination, which is known to be robustly processed by 120 hpf [32]

### 2.5. Gene Expression Analysis 

To analyze changes in gene expression profiles upon exposure to BBzP, DEHP, and DiNP, zebrafish embryos were exposed to four concentrations (i.e., 0, 5, 50, and 500 µg/L) from 2 hpf to 120 hpf and 20 whole zebrafish larvae were pooled at 120 hpf. Three replicates were employed for each concentration. The expression levels of four transcription factors (i.e., *ache dat*, *drd1b*, and *th*) that are potentially involved in production and function of neurochemicals were measured. Primers were designed using the Primer-BLAST tool (https://www.ncbi.nlm.nih.gov/tools/primer-blast/) or the PrimerQuest tool (https://eu.idtdna.com/pages/tools/primerquest). The primer sequences are listed in Table S1. In brief, total RNA was extracted using RNAzol reagent (Sigma-Aldrich) and reverse-transcribed into complementary DNA (cDNA) using the Applied Biosystems™ High-Capacity cDNA Reverse Transcription Kit (Thermo Fisher Scientific, Waltham, MA, USA). Gene expression was quantified by polymerase chain reaction (PCR) using LightCycler^®^ 96 Real-Time PCR System (Roche Life Science, Penzberg, Germany). Each PCR reaction comprised 2 μL of diluted cDNA, 0.6 μL of appropriate forward and reverse primers, 10 μL of FastStart Essential DNA Green Master reaction mix (Roche Life Science), and 6.8 μL of PCR-grade water (Roche Life Science). The fold change in gene expression was calculated using the 2^−∆∆Ct^ method and normalized to that of *β-actin*, as an internal housekeeping gene [33]. Beta-actin was determined as an internal house-keeping gene after the quality and stability were confirmed and compared with other four housekeeping genes according to the BestKeeper protocol [34]. As shown in Table S2, all of them met the requirement for the minimum standard deviation (SD) of the cycle of quantification (Cq) values. Beta-actin was proven the most stable with respect to small SD and coefficient of variation of Cq values in both untreated control and exposed groups.

### 2.6. Statistical Analysis

Statistical analyses of body length, eye size, FI, and gene expression were performed using one-way analysis of variance (ANOVA) followed by Dunnett’s post-hoc test with Sigma Plot 13.0 software (Systat Software, Inc., San Jose, CA, USA). Values are expressed as the mean ± standard error of the mean (SEM). A probability (*p*) value of < 0.05 was considered statistically significant. Significance is denoted by asterisks (* *p* < 0.05, ** *p* < 0.01). 

## 3. Results

### 3.1. Developmental Toxicity Profiles of Phthalates

The incidence of embryonic mortality and malformation upon exposure to the six tested PAEs was shown in Figure S1. Mortality was observed only at the highest concentration (100 K μg/L) of DMP, DEP, and BBzP. Exposure to DEHP, DnOP, and DiNP did not lead to mortality even at the highest concentration. DMP and DEP were the most toxic (100% mortality at 100 K μg/L). However, lethality was observed at 24 hpf and 120 hpf for DEP and DMP, respectively. The incidence of malformation was similar to the level observed in the control group (<0%) for all PAEs at all tested concentrations. The analysis of body length and eye size diameter at 120 hpf, revealed toxicity only at the highest concentration (100 K). Body length was significantly affected by BBzP and DEHP, while eye size was significantly reduced by BBzP and DnOP (Table S3).

### 3.2. Neurobehavioral Locomotor Response 

**Figure 1:**
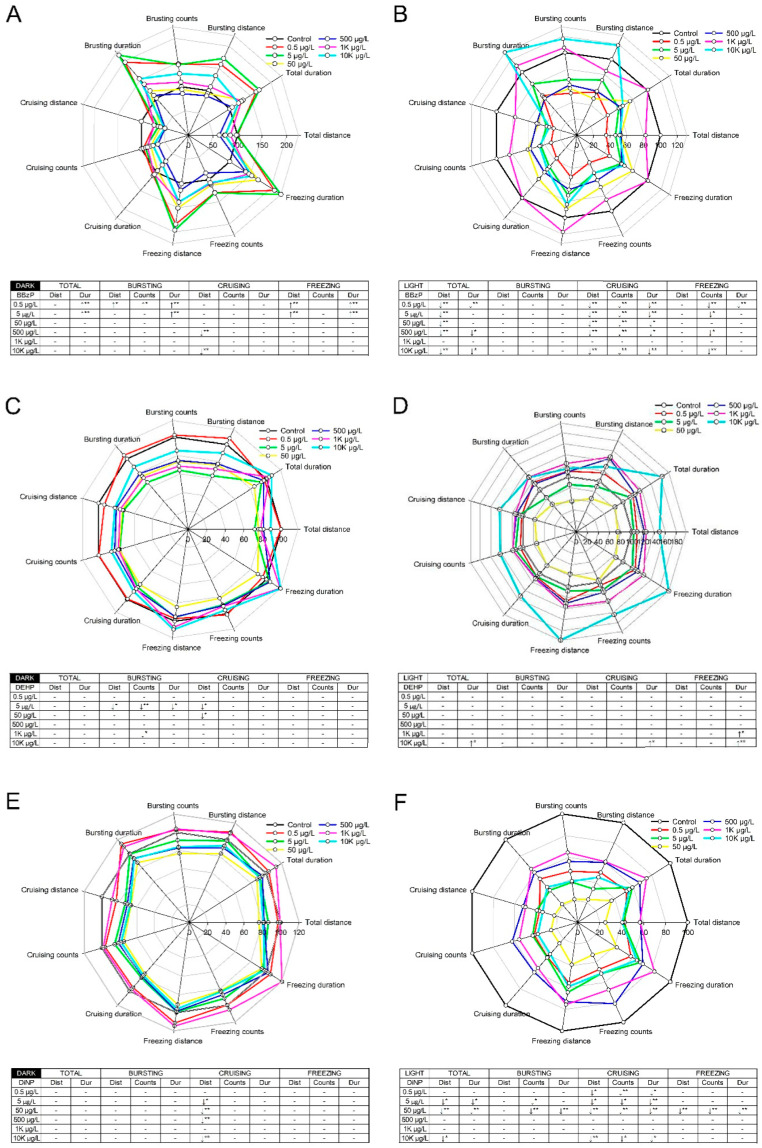
Locomotor responses of larval zebrafish (*n* = 48) upon exposure to diisononyl phthalate (BBzP) (**A**,**B**), di-2-ethylhexyl phthalate (DEHP) (**C**,**D**), and benzyl butyl phthalate (DiNP) (**E**,**F**). Locomotor response was divided into the dark (**A**,**C**,**E**) and light phase (**B**,**D**,**F**) for each phthalate. (↑) represents a significant hyperactivity compared with control and (↓) indicates a significant hypoactivity compared with control. (* *p* < 0.05; ** *p* < 0.01). Dist: distance. Dur: duration. The attention is needed on different scale of total distance in each graph.

Swimming behavior was evaluated under alternating light–dark conditions using a locomotor response assay. There were no significant differences in behavioral activities upon exposure to DMP, DEP, and DnOP (Figure S2). In contrast, BBzP, DEHP, and DiNP elicited differential light and dark responses in zebrafish larvae. Compared with the control group, swimming, bursting, and freezing activities were significantly increased by treatment in the dark phase with BBzP at 0.5 μg/L and 5 μg/L, while cruising duration was significantly decreased at concentrations of 500 μg/L and 10 K μg/L. In the light phase, larval activity was decreased significantly with respect to total swimming, cruising, and freezing activities at all concentrations with the exception of 1 K μg/L (Figure 1A,B). DEHP caused minor changes in swimming activity, as compared to the control group; however, these changes were not concentration dependent. In the dark phase, larval zebrafish exhibited hyperactivity at high concentrations (1 K μg/L and 10 K μg/L) of DEHP (Figure 1C,D). Upon exposure to DiNP, cruising activity was inhibited in the light phase, and hypoactivity was observed in bursting, cruising, and freezing activities in the dark phase. Notably, the behavioral effects elicited by DiNP were observed at low concentrations (0.5, 5, and 50 μg/L) (Figure 1E,F).

### 3.3. Changes in Fluorescence Intensity of Three Tg Lines

**Figure 2:**
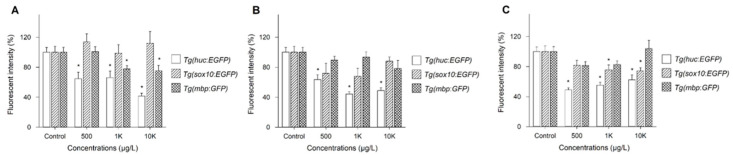
FIs of *Tg(huc*:*EGFP)*, *Tg(sox10*:*EGFP)*, and *Tg(mbp*:*GFP)* for BBzP (**A**), DEHP (**B**), and DiNP (**C**). FI was averaged from 10 images/treatment. Values are expressed as the mean ± SEM. * *p* < 0.05.

**Figure 3:**
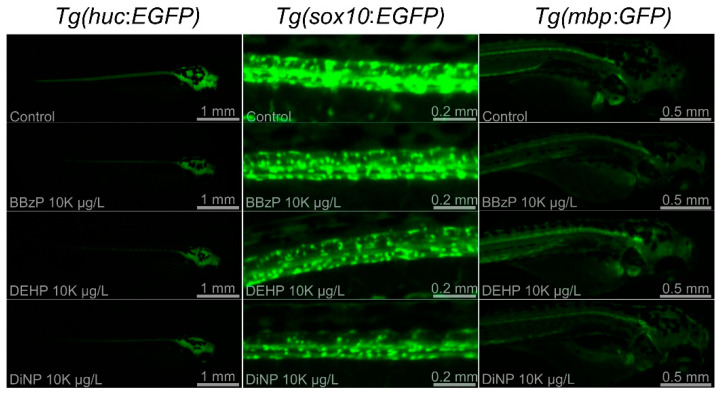
Representative images of *Tg(HuC*:*EGFP)*, *Tg(sox10*:*EGFP)*, and *Tg(mbp*:*GFP)* in untreated controls and embryos treated with BBzP, DEHP, and DiNP at 10 K μg/L.

Three Tg lines, *Tg(HuC*:*eGFP)*, *Tg(sox10*:*eGFP)*, and *Tg(mbp*:*GFP)*, were employed to investigate the mechanisms underlying neurotoxicity upon exposure to three phthalates showing abnormal neurobehavior: BBzP, DEHP, and DiNP. Because no significant changes were observed at 5 μg/L and 50 μg/L, the results on FI were shown at 500 μg/L, 1 K μg/L, and 10 K μg/L. Changes in FI of three Tg lines were dependent on PAEs (Figure 2). For BBzP, the FI of *Tg(sox10:eGFP)* was not affected, while that of *Tg(HuC*:*eGFP)* and *Tg(mbp*:*GFP)* was reduced. Notably, the reduction in FI upon exposure to *Tg(HuC*:*eGFP)* and *Tg(mbp*:*GFP)* was concentration-dependent. For DEHP, FI was reduced only in *Tg(HuC*:*eGFP)*. Lastly, for DiNP, FI of *Tg(HuC*:*eGFP)* and *Tg(sox10:eGFP)* was reduced, while no effect was observed in *Tg(mbp*:*GFP)*. Reduction in FI of *Tg(HuC*:*eGFP)* was observed at all concentrations. Representative images are shown in Figure 3.

### 3.4. Changes in Expression Profiles of Genes Involved in Neurotransmission

**Figure 4:**
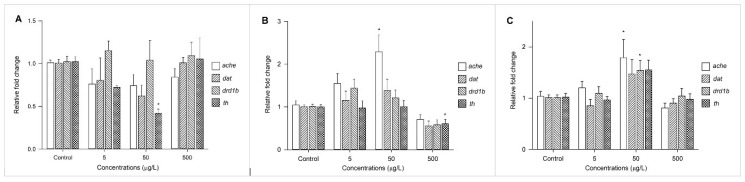
Fold change to expression levels of *ache*, *dat*, *drd1b*, and *th* in zebrafish larvae exposed to BBzP (**A**), DEHP (**B**), and DiNP (**C**) at concentrations of 5, 50, and 500 μg/L. Values are expressed as the mean ± SEM. * *p* < 0.05.

Changes in the expression profiles of acetylcholinesterase (*ache*) and three transcription factors (i.e., *dat*, *drd1b*, and *th*) involved in the dopamine system were measured after exposure to BBzP, DEHP, and DiNP at concentrations of 5 μg/L, 50 μg/L, and 500 μg/L (Figure 4). The expression of *ache* was upregulated by exposure to DiNP and DEHP, and the expression *drd1b* was upregulated by DiNP. Finally, the expression of *th* was suppressed by BBzP and DEHP. 

## 4. Discussion

The six tested PAEs (i.e., DMP, DEP, BBzP, DnOP, DEHP, and DiNP) showed low developmental toxicity in zebrafish embryos with respect to embryonic mortality and malformation. Exposure to PAEs induced no consistent or concentration-dependent malformations and embryonic mortality was observed only at the highest concentration (0.1 g/L) of DMP, DEP, and BBzP. PAEs with low molecular weights (P-LMWs) were more toxic than those with high molecular weights (P-HMWs) with a backbone of >6 carbon atoms. In other studies, P-LMWs were toxic compared with P-HMWs [35,36]. 

Exposure to BBzP, DEHP, and DiNP altered the behavioral activities of zebrafish larvae whereas DMP, DEP, and DnOP had no effects. In previous studies, the differential influence of PAEs (i.e., DEHP and DBP) on behavioral activities was observed even in studies that employed zebrafish embryos [37,38,39,40,41]. For example, exposure to DEHP resulted in abnormal locomotive activity [38], in contrast, no change in locomotive behavior was reported in Dach et al. (2019) [37]. Experimental conditions, such as the presence of a chorion and exposure concentration or window may contribute to these different outcomes [22,42]. In particular, in the present study, BBzP and DiNP had the greatest effects on speed (i.e., bursting, cruising, and freezing) and movement parameters (i.e., distance, count, and duration), whereas changes in behavioral activities were intermittent in the DEHP-exposed group. Although the relationship between changes in speed-related movement and hypo- or hyperactivities of zebrafish is largely unknown, bursting is associated with avoidance movements [43] and axons controlling the avoidance escape circuits from hazards are the largest in the animal central neural cord [44]. Moreover, cruising is associated with stable movements [36], which are regulated, at least in part, by subcortical structures [45]. Finally, freezing is strongly dependent on the periaqueductal gray components of the amygdala [46,47,48]. These findings suggest that behavioral alterations induced by three PAEs (i.e., BBzP, DEHP, and DiNP) could be attributed to negative impacts on the brain and CNS of developing zebrafish embryos. 

Exposure to BBzP and DiNP induced greater effects on behavior activity in the light phase than in the dark phase; hypoactivity was observed when there was a sudden increase in light. Changes in locomotor activity in the light phase have also been reported from exposure to other chemicals (i.e., picrotoxin, eliprodil, and acrylamide) [49,50]. Fernandes et al. (2012) demonstrated that the locomotor response to light was controlled by brain photoreceptors and mediated with sensory neurons [51], and Emran et al. (2007) reported that ON–OFF function in retinal neurons was associated with the response of larvae to alternating light intensity [52]. These observations suggest that visual impairment partially contributed to abnormal neurobehavior in the light phase upon exposure to BBzP and DiNP. In this study, BBzP exposure decreased diameter of the eye size (Table S3). In amphibians and rats, previous studies observed malformations in the eye upon exposure to phthalates such as DEP, dibutyl phthalate, and DEHP [53,54]. More studies are required to identify the causality between abnormal neurobehavior in the light phase caused by BBzP or DiNP exposure and damage to the visual system.

Changes in FI in the three Tg lines varied with DiNP, BBzP, and DEHP. A reduced expression of *elavl3* was observed by exposure to all three PAEs, indicating that exposure to DiNP, BBzP, and DEHP inhibited the activities of the primary neurons during the early developmental stage. Previous studies reported the use of *Tg(HuC*:*eGFP)* to assess alterations in the primary neuron activities after the gastrulation stage [30,55]. Chen et al. (2012) reported that reduced behavior in zebrafish exposed to 2,2′,4,4′-tetrabromodiphenyl ether (BDE-47) was attributable to dysfunction of the primary neurons [56]. Moreover, *sox10* expression was decreased in the DiNP-exposed groups, which could explain the observed hypoactivity. *Tg(sox10*:*eGFP)* represents the expression of neural crest cells and optic placode in zebrafish [57,58,59]. Kim et al. (2013) reported the relationship between decreased *sox10* expression and hypoactivity in zebrafish exposed to gold nanoparticles [29]. The present study also observed that only BBzP reduced the intensity of FI in *Tg(mbp*:*GFP)* zebrafish. For BBzP, both hyperactivity and hypoactivity were observed in the dark and light phases. As described above, hypoactivity could be caused by the reduced expression of *elavl3* in *Tg(HuC*:*eGFP)*. However, information on hyperactivity or concurrent hypo- and hyperactivity is currently limited. Zada et al. (2014) demonstrated that mutations to monocarboxylate transporter 8 (mct8−/−) altered the response to transitional light and dark conditions, and showed that the reduction in *mbp* expression was accompanied [60]. Hence, further studies are necessary to investigate alterations in different behavioral activities upon exposure to BBzP in alternating light and dark phases. Taken together, these findings indicate that the underlying mechanisms of neurotoxicity vary among different PAEs. 

Changes in the expression patterns of genes involved in the cholinergic (ac*he)* and dopaminergic systems (i.e., *dat*, *drd1b*, and *th*) varied among the individual PAEs. Previous studies reported that *ache* and dopamine D1 receptors (regulated by *drd1b*) play critical roles in the neuromuscular system and behavioral activities [61,62], and that the inhibition of *th* expression is associated with neural diseases, such as Parkinsonism [63,64]. It appears that alterations in behavioral activities by exposure to PAEs are accompanied by the production and function of neurochemicals. However, further studies are necessary to clarify the entangled mechanisms underlying neurotoxicity due to individual PAEs.

## 5. Conclusions

Exposure to BBzP, DEHP, and DiNP resulted in disordered swimming behavior, and BBzP was the most neurotoxic to zebrafish embryos. Damage to the primary neurons and the reduced expression of genes associated with CNS development were responsible for changes in behavioral activities, thereby linking alterations in neurotransmission systems. However, more mechanistic studies are needed on the causality between varying phenotypic behavior alterations (i.e., hypo- and hyperactivity) and changes at the molecular level.

## Data Availability

The data presented in this study are openly available.

## Supplementary Materials

The following are available online at https://www.mdpi.com/2305-6304/9/1/5/s1, Table S1: List of the primers used for real-time polymerase chain reaction analysis. Table S2: Quality and stability of housekeeping genes in untreated control and exposed groups. Table S3: Body length and eye size of zebrafish larvae treated with six phthalates. The values are expressed as the mean ± standard error of the mean. * p < 0.05. Figure S1: Developmental toxicity of six phthalates: dimethyl phthalate (DMP), diethyl phthalate (DEP), benzyl butyl phthalate (BBzP), di-2-ethylhexyl phthalate (DEHP), di-n-octyl phthalate (DnOP), and diisononyl phthalate (DiNP). Survival rate (A) and malformation rate (B) at different concentrations in zebrafish larvae (n = 48) after five days of exposure. Figure S2: Locomotor responses of larval zebrafish (n = 48) upon exposure to dimethyl phthalate (DMP) (A,B), diethyl phthalate (DEP) (C,D), and di-n-octyl phthalate (DnOP) (E,F). Locomotor response was divided into the dark (A,C,E) and light phase (B,D,F) for each phthalate. (↑) represents a significant hyperactivity compared to control and (↓) indicates a significant hypoactivity in compared to control. (* *p* < 0.05; ** *p* < 0.01). Dist: distance. Dur: duration.
