# Supplementary material for: Comparative Analysis of Neurotoxicity of Six Phthalates in Zebrafish Embryos
Source: Toxics. 2021 Jan 7;9(1):5. doi: 10.3390/toxics9010005 (PMC7825694; doi:10.3390/toxics9010005)
Supplement: Supplementary file 1 [file toxics-09-00005-s001.pdf]

Supplementary Materials: Comparative Analysis of Neurotoxicity of Six Phthalates in Zebrafish Embryos

Cong Minh Tran, Trinh Ngoc Do and Ki-Tae Kim

Table S1. List of the primers used for real-time polymerase chain reaction analysis.

Symbol	Gene Name	Forward Primer (5' to 3')	Reverse Primer (5' to 3')	Ncbi Accession #
β-actin	beta-actin 2	CGAGCTGTCTTCCCATCCA	TCACCAACGTAGCTGTCTTTCTG	NM_181601.5
ache	Acetylcholinesterase	TCCAAGTCAGTGCTGTGATAAG	GGAAGAGATAAGTGGGCAAAGA	NM_131846.2
dat	dopamine transporter	GCTGGAAATTTGTGAGCCCG	AGGAACCGTACTTGGGAGGA	NM_131755.1
th1	tyrosine hydroxylase	ACCAAAGGATGGCTTGGAGG	CGTGCTAACATCCGACAGGT	NM_131149.1
drd1b	dopamine receptor D1	TCACCTTCCATGTCAGGCAC	GAGTCAGATCTCACCCGCTG	NM_001135976.2

Table S2. Quality and stability of housekeeping genes in untreated control and exposed groups.

Cq Values	Control Group					Exposed Group				
**Gene names**	***beta-actin***	***elfa***	***gapdh***	***tuba1***	***tbp***	***beta-actin***	***elfa***	***gapdh***	***tuba1***	***tbp***
**n**	9	9	9	9	9	9	9	9	9	9
**geo Mean [Cq]**	16.392826	15.54283	18.3031	23.49121	18.53468	15.3669993	16.18581	19.29837	19.29145	23.94078
**ar Mean [Cq]**	16.393333	15.54333	18.30333	23.49222	18.53889	15.3677778	16.18778	19.29889	19.29222	23.94111
**min [Cq]**	16.17	15.36	18.13	23.29	17.99	15.08	15.88	19.04	19.07	23.73
**max [Cq]**	16.54	15.85	18.43	23.98	19.19	15.59	16.65	19.53	19.55	24.12
**SD [± Cq]**	0.1133333	0.083704	0.078519	0.171852	0.36321	0.12592593	0.225926	0.119012	0.157531	0.109877
**CV [% Cq]**	0.6913379	0.538518	0.428985	0.731527	1.959178	0.81941532	1.395657	0.61668	0.816551	0.458945
**min [x-fold]**	−1.167018	−1.13511	−1.12748	−1.14966	−1.45869	−1.2200999	−1.23612	−1.19613	−1.1659	−1.15731
**max [x-fold]**	1.1073979	1.237276	1.091946	1.40327	1.57497	1.16715865	1.37954	1.174159	1.196279	1.13227
**std dev [± x-fold]**	1.0817247	1.059735	1.055933	1.126504	1.286285	1.09120785	1.169528	1.085991	1.115377	1.079136

Cq: the cycle of quantification values; SD: standard deviation; CV: coefficient of variation.

Table S3. Body length and eye size of zebrafish larvae treated with six phthalates. The values are expressed as the mean ± SD. *$p < 0.05$.

Concentration (μ g/L)		0	0.5	5	50	500	1K	10K	100K
**Body Length (mm)**	**DMP**	3.67 ± 0.06	3.68 ± 0.04	3.71 ± 0.1	3.7 ± 0.12	3.67 ± 0.17	3.75 ± 0.11	3.79 ± 0.1	NM
	**DEP**	3.73 ± 0.05	3.77 ± 0.1	3.7 ± 0.15	3.65 ± 0.05	3.76 ± 0.07	3.69 ± 0.13	3.78 ± 0.11	NM
	**BBzP**	3.87 ± 0.11	3.66 ± 0.11	3.66 ± 0.19	3.64 ± 0.11*	3.58 ± 0.18*	3.64 ± 0.14*	3.79 ± 0.14	3.41 ± 0.25*
	**DEHP**	3.74 ± 0.1	3.74 ± 0.15	3.78 ± 0.1	3.73 ± 0.09	3.85 ± 0.09	3.86 ± 0.09	3.87 ± 0.14	3.93 ± 0.07*
	**DnOP**	3.74 ± 0.11	3.74 ± 0.07	3.75 ± 0.1	3.73 ± 0.11	3.73 ± 0.09	3.79 ± 0.11	3.78 ± 0.1	3.7 ± 0.17
	**DiNP**	3.78 ± 0.09	3.59 ± 0.07	3.83 ± 0.08	3.81 ± 0.15	3.63 ± 0.1	3.84 ± 0.09	3.81 ± 0.1	3.75 ± 0.1
**Eye Size (μm)**	**DMP**	329.06 ± 10.68	330.17 ± 9.14	325.39 ± 12.79	331.09 ± 6.73	320.31 ± 28.48	309.73 ± 12.52	323.24 ± 10.9	NM
	**DEP**	324.47 ± 6.72	333.28 ± 13.06	329.23 ± 16.04	321.34 ± 12.74	323.71 ± 5.86	319.6 ± 16.18	311.09 ± 5.97	NM
	**BBzP**	331.91 ± 12.96	320.93 ± 5.49	313.59 ± 23.67	321.98 ± 9.19	310.08 ± 17.18	317.96 ± 9.65	315.41 ± 12.79	267.57 ± 34.11*
	**DEHP**	340.72 ± 11.54	331.29 ± 11.95	337.98 ± 9.32	340.27 ± 7.71	340.65 ± 6.65	341.15 ± 10.08	336.61 ± 14.59	344.17 ± 5.43
	**DnOP**	322.66 ± 7.31	329.7 ± 8.18	329.41 ± 9.13	326.72 ± 9.59	329.82 ± 12.8	329.38 ± 8.52	330.02 ± 14.75	303.44 ± 13.77*
	**DiNP**	334.63 ± 7.78	327.06 ± 5.67	333.4 ± 8.47	330.05 ± 4.67	334.03 ± 22.07	335.19 ± 7.19	334.29 ± 4.59	334.35 ± 10.95

[a] NM: Not measured.

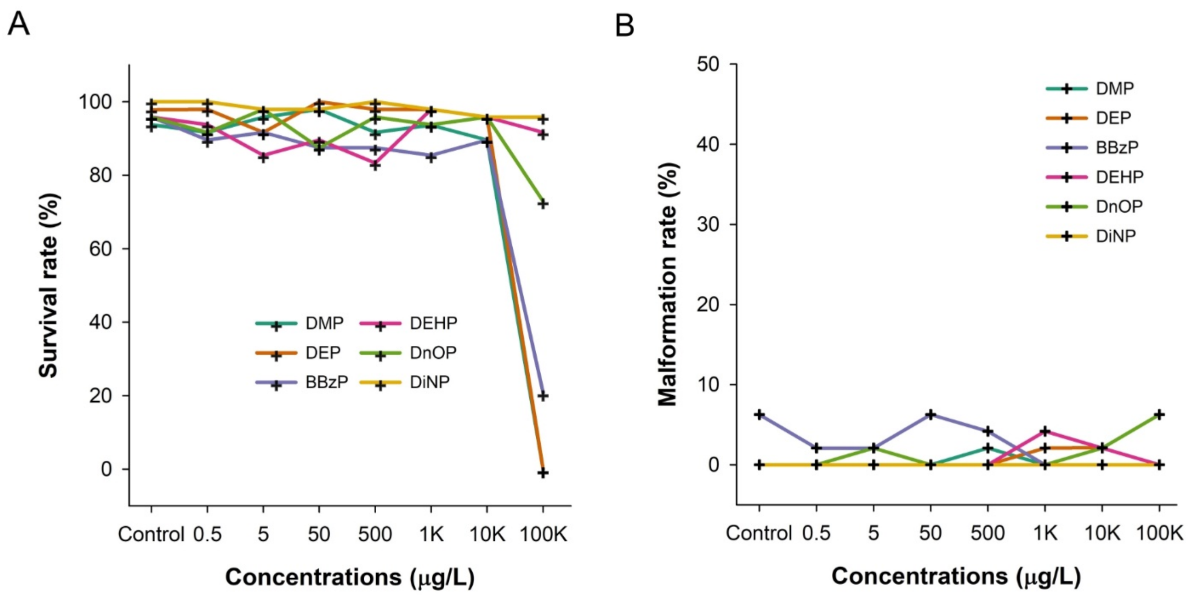

Figure S1. Developmental toxicity of six phthalates: dimethyl phthalate (DMP), diethyl phthalate (DEP), benzyl butyl phthalate (BBzP), di-2-ethylhexyl phthalate (DEHP), di-n-octyl phthalate (DnOP), and diisononyl phthalate (DiNP). Survival rate (**A**) and malformation rate (**B**) at different concentrations in zebrafish larvae ($n = 48$) after five days of exposure.

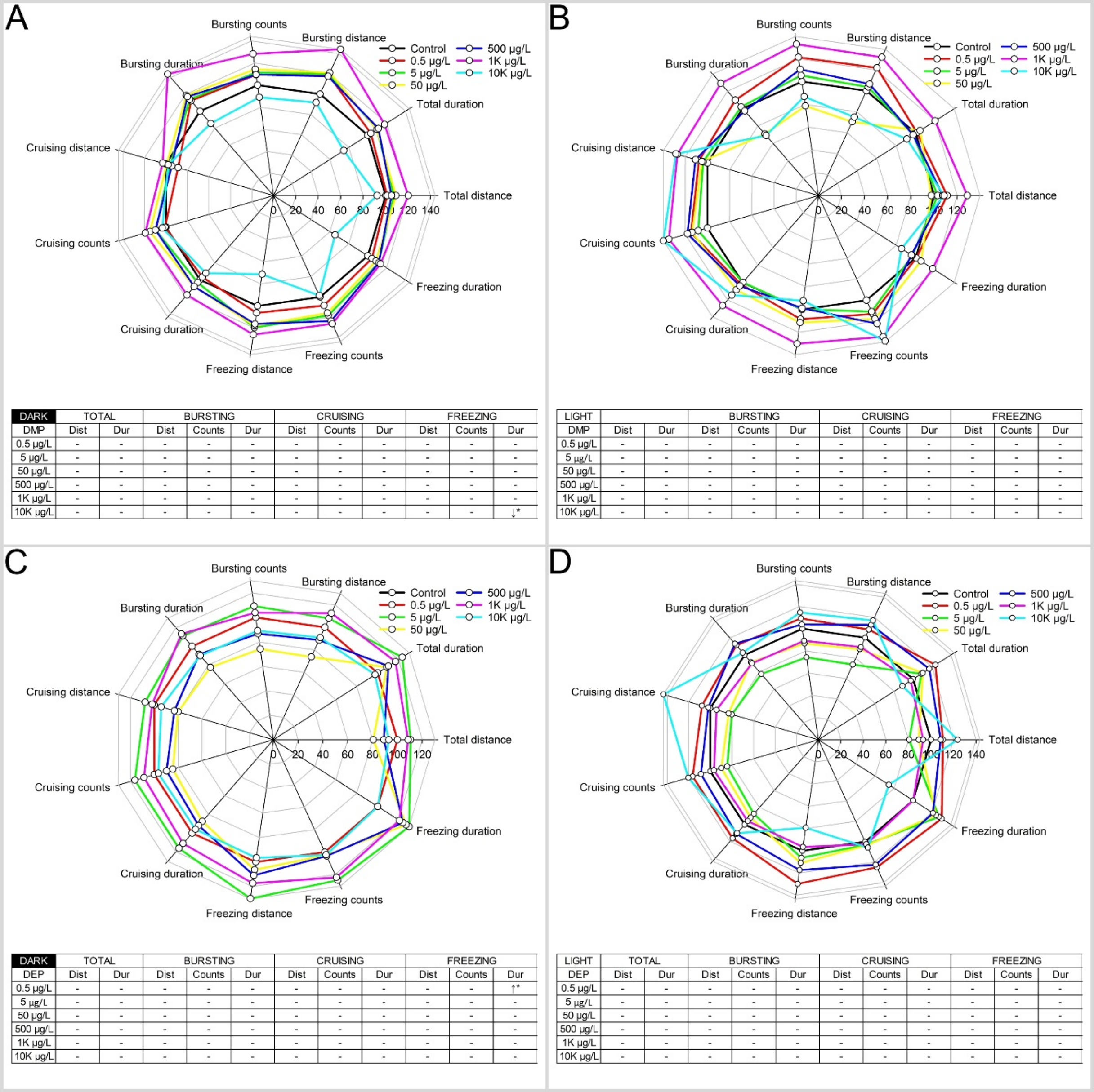

DARK	TOTAL		BURSTING			CRUISING			FREEZING		
DMP	Dist	Dur	Dist	Counts	Dur	Dist	Counts	Dur	Dist	Counts	Dur
0.5 µg/L	-	-	-	-	-	-	-	-	-	-	-
5 µg/L	-	-	-	-	-	-	-	-	-	-	-
50 µg/L	-	-	-	-	-	-	-	-	-	-	-
500 µg/L	-	-	-	-	-	-	-	-	-	-	-
1K µg/L	-	-	-	-	-	-	-	-	-	-	-
10K µg/L	-	-	-	-	-	-	-	-	-	-	↓*

LIGHT			BURSTING			CRUISING			FREEZING		
DMP	Dist	Dur	Dist	Counts	Dur	Dist	Counts	Dur	Dist	Counts	Dur
0.5 µg/L	-	-	-	-	-	-	-	-	-	-	-
5 µg/L	-	-	-	-	-	-	-	-	-	-	-
50 µg/L	-	-	-	-	-	-	-	-	-	-	-
500 µg/L	-	-	-	-	-	-	-	-	-	-	-
1K µg/L	-	-	-	-	-	-	-	-	-	-	-
10K µg/L	-	-	-	-	-	-	-	-	-	-	-

DARK	TOTAL		BURSTING			CRUISING			FREEZING		
DEP	Dist	Dur	Dist	Counts	Dur	Dist	Counts	Dur	Dist	Counts	Dur
0.5 µg/L	-	-	-	-	-	-	-	-	-	-	↑*
5 µg/L	-	-	-	-	-	-	-	-	-	-	-
50 µg/L	-	-	-	-	-	-	-	-	-	-	-
500 µg/L	-	-	-	-	-	-	-	-	-	-	-
1K µg/L	-	-	-	-	-	-	-	-	-	-	-
10K µg/L	-	-	-	-	-	-	-	-	-	-	-

LIGHT	TOTAL		BURSTING			CRUISING			FREEZING		
DEP	Dist	Dur	Dist	Counts	Dur	Dist	Counts	Dur	Dist	Counts	Dur
0.5 µg/L	-	-	-	-	-	-	-	-	-	-	-
5 µg/L	-	-	-	-	-	-	-	-	-	-	-
50 µg/L	-	-	-	-	-	-	-	-	-	-	-
500 µg/L	-	-	-	-	-	-	-	-	-	-	-
1K µg/L	-	-	-	-	-	-	-	-	-	-	-
10K µg/L	-	-	-	-	-	-	-	-	-	-	-

Figure S2. Locomotor responses of larval zebrafish ($n = 48$) upon exposure to dimethyl phthalate (DMP) (A, B), diethyl phthalate (DEP) (C, D), and di-n-octyl phthalate (DnOP) (E, F). Locomotor response was divided into the dark (A, C, E) and light phase (B, D, F) for each phthalate. (↑) represents a significant hyperactivity compared to control and (↓) indicates a significant hypoactivity in compared to control. (*$p < 0.05$; **$p < 0.01$). Dist: distance. Dur: duration.

E

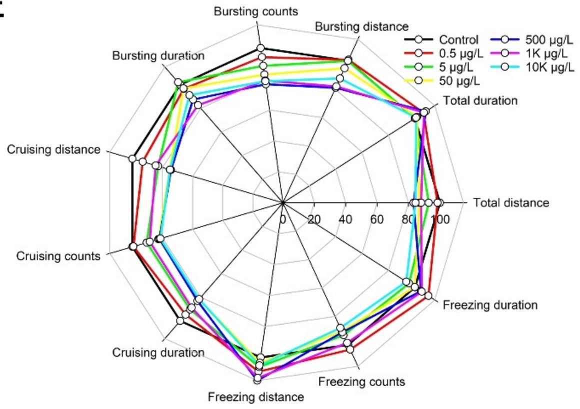

DARK	TOTAL		BURSTING			CRUISING			FREEZING		
DnOP	Dist	Dur	Dist	Counts	Dur	Dist	Counts	Dur	Dist	Counts	Dur
0.5 μg/L	-	-	-	-	-	-	-	-	-	-	-
5 μg/L	-	-	-	-	-	-	-	-	-	-	-
50 μg/L	-	-	-	-	-	-	-	-	-	-	-
500 μg/L	-	-	-	-	-	-	-	-	-	-	-
1K μg/L	-	-	-	-	-	-	-	-	-	-	-
10K μg/L	-	-	-	-	-	-	-	-	-	-	-

F

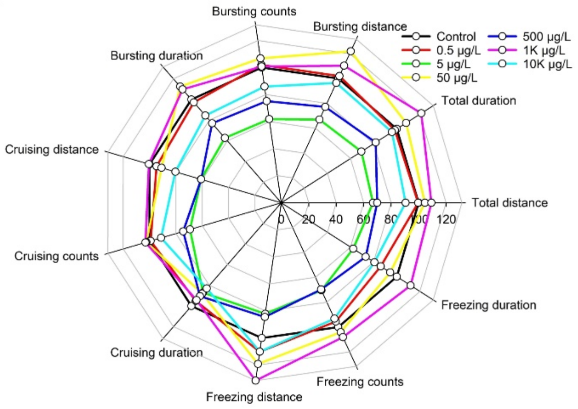

LIGHT	TOTAL		BURSTING			CRUISING			FREEZING		
DnOP	Dist	Dur	Dist	Counts	Dur	Dist	Counts	Dur	Dist	Counts	Dur
0.5 μg/L	-	-	-	-	-	-	-	-	-	-	-
5 μg/L	-	-	-	-	-	-	-	-	-	-	-
50 μg/L	-	-	-	-	-	-	-	-	-	-	-
500 μg/L	-	-	-	-	-	-	-	-	-	-	-
1K μg/L	-	-	-	-	-	-	-	-	-	-	-
10K μg/L	-	-	-	-	-	-	-	-	-	-	-

(continued) Figure S2. Locomotor responses of larval zebrafish (n = 48) upon exposure to dimethyl phthalate (DMP) (A, B), diethyl phthalate (DEP) (C, D), and di-n-octyl phthalate (DnOP) (E, F). Locomotor response was divided into the dark (A, C, E) and light phase (B, D, F) for each phthalate. (↑) represents a significant hyperactivity compared to control and (↓) indicates a significant hypoactivity in compared to control. (*p < 0.05; **p < 0.01). Dist: distance. Dur: duration.
